# Are There Gender Differences in Emotion Comprehension? Analysis of the Test of Emotion Comprehension

**DOI:** 10.1007/s10826-017-0956-5

**Published:** 2017-12-11

**Authors:** Angel M. Fidalgo, Harriet R. Tenenbaum, Ana Aznar

**Affiliations:** 1 University of Oviedo, Oviedo, Spain; 2 University of Surrey, Guildford, UK; 3 University of Winchester, Winchester, UK

**Keywords:** Emotion understanding, Test of Emotion Comprehension, Gender differences, Differential item functionin, False belief task

## Abstract

This article examines whether there are gender differences in understanding the emotions evaluated by the Test of Emotion Comprehension (TEC). The TEC provides a global index of emotion comprehension in children 3–11 years of age, which is the sum of the nine components that constitute emotion comprehension: (1) recognition of facial expressions, (2) understanding of external causes of emotions, (3) understanding of desire-based emotions, (4) understanding of belief-based emotions, (5) understanding of the influence of a reminder on present emotional states, (6) understanding of the possibility to regulate emotional states, (7) understanding of the possibility of hiding emotional states, (8) understanding of mixed emotions, and (9) understanding of moral emotions. We used the answers to the TEC given by 172 English girls and 181 boys from 3 to 8 years of age. First, the nine components into which the TEC is subdivided were analysed for differential item functioning (DIF), taking gender as the grouping variable. To evaluate DIF, the Mantel–Haenszel method and logistic regression analysis were used applying the Educational Testing Service DIF classification criteria. The results show that the TEC did not display gender DIF. Second, when absence of DIF had been corroborated, it was analysed for differences between boys and girls in the total TEC score and its components controlling for age. Our data are compatible with the hypothesis of independence between gender and level of comprehension in 8 of the 9 components of the TEC. Several hypotheses are discussed that could explain the differences found between boys and girls in the belief component. Given that the Belief component is basically a false belief task, the differences found seem to support findings in the literature indicating that girls perform better on this task

## Introduction

Emotion understanding is an ability that refers to the way in which individuals understand, predict, and explain the feelings of others and oneself (Denham 1998; Harris 1989; Saarni 1999). Children with a good level of emotion understanding are more popular among their peers, have more friends (Denham et al. 1990), do better academically (Izard et al. 2001), and show lower levels of psychological problems, such as depression, bipolar disorder, and schizophrenia (for a review see Cicchetti et al. 1995) than children who have lower levels of emotion understanding.

Children undergo three basic levels of cognitive emotion understanding (Pons et al. 2004). From the ages of 3–5 years, children gain an understanding of external aspects of emotions such as learning to recognize facial expressions of emotions. From the ages of 5–7 years, children acquire a mentalistic emotion understanding. For children to acquire a mentalistic emotion understanding, they must develop a theory of mind (ToM), which is the ability to understand that others have thoughts and beliefs that differ from one’s own. Mentalistic emotion understanding includes emotions resulting from beliefs and desires. Finally, between the ages of 7 and 9 years, children understand that we can reflect on a situation from different perspectives (Pons et al. 2004).

Although children’s development of emotion understanding undergoes a specific developmental pattern, there are individual differences in children’s emotion understanding using different tests, such as the Test of Emotion Comprehension (TEC; Pons and Harris 2005) and Denham’s Emotion Understanding Test (Denham 1986; Martin and Green 2005). There are a number of factors (e.g., mothers’ emotion talk, children’s language skills) that predict these individual differences. One such factor is children’s gender (Fivush et al. 2000).

Much research has been devoted to understanding whether there are gender differences in emotion understanding. Many studies have found that girls tend to have a better emotion understanding than boys (Bosacki and Moore 2004 with a puppet task based on Capps et al. 1992; Brown and Dunn 1996 and Denham and Kochanoff 2002, based on Denham’s (1986) Affect Knowledge Test (AKT); Garner and Waajid 2008, based on a vignette-based task designed by Michalson and Lewis 1985). A few studies have found that boys score higher than girls on emotion understanding (Laible and Thompson 2000 with measures based on Denham’s (1986) AKT). Even more studies do not find gender differences in emotion understanding (Albanese et al. 2006 with the TEC, Bennett et al. 2005 with vignettes based on Michalson and Lewis 1985; Denham et al. 2012 and Hughes and Dunn 1998 with measures based on Denham’s (1986) AKT; Pons et al. 2004 with the TEC).

Part of the reason differences may not be found is that when measures of emotion understanding are aggregated across different aspects of emotion understanding, it may mask gender differences in specific areas. For example, Aznar and Tenenbaum (2013) found no gender differences between 4-year-old children in overall emotion understanding as assessed by the TEC. However, 6-year-old boys scored higher than 6-year-old girls in understanding the situational causes of emotion, whereas 6-year-old girls scored higher on understanding reflective emotions than did 6-year-olds boys. Thus, it seems that girls and boys might differ from each other in different types of emotion understanding at particular ages.

The TEC provides a global index of emotion comprehension in children 3 to 11 years of age, which is the sum of the nine components that constitute emotion comprehension: (1) recognition of facial expressions, (2) understanding of external causes of emotions, (3) understanding of desire-based emotions, (4) understanding of belief-based emotions, (5) understanding of the influence of a reminder on present emotional states, (6) understanding of the possibility to regulate emotional states, (7) understanding of the possibility of hiding emotional states, (8) understanding of mixed emotions, and (9) understanding of moral emotions (for a detailed description of the test, see (Francisco Pons et al. 2004).

From a psychometric viewpoint, the TEC is a reliable and valid instrument as shown by studies conducted to date. Thus, Pons et al. (2002) report a good test–retest reliability after 3-months (*r* (18) = .84) and Pons and Harris (2005) a good test-retest correlation after a 13-month delay (*r* (40) = .64 and *r* (32) = .54). When internal consistency was used as a measure of reliability using Cronbach’s alpha all the values are in the range of .61 to .97; Albanese and Molina (2008), *α* = .79; Farina and Belacchi (2014), *α* = .76; Karstad et al. (2014), *α* = .61.

It should be noted that when items are not strictly parallel, or are dichotomous, the Cronbach’s coefficient provides a lower-bound estimate of true reliability. For this reason, some authors have used the theta and phi-coefficients to estimate the internal consistency reliability. Both coefficients provide an estimate of the maximum value of Cronbach’s coefficient alpha (Gadermann et al. 2008; Sun et al. 2007). Thus, Karstad et al. (2015), using the theta test to assess the reliability, obtained values of .82 and .91, and Karstad et al. (2014) obtain a value of .95 using the phi-coefficient. Previous studies have shown that the nine components of the TEC meet the requirements for a Guttman scale. This means that the components of the TEC form an ordinal scale which can be ordered hierarchically in such a way that correctly responding to one component also implies a correct response to lower-order components. The scale is usually considered valid when the coefficient of reproducibility is over 0.9 and the consistency index is over 0.5. Both indices show to what extent the items form a perfect scale (Green 1956). Pons et al. (2004) found values of 0.904 and 0.68 in the reproducibility coefficient and the consistency index, respectively. Mokken scale analysis of TEC components also yielded satisfactory results (*H* = 0.40, *Rho* = 0.79; Albanese and Molina (2008)). Furthermore, evidence of their criterion validity can be found in Albanese and Molina (2008), and Pons et al. (2014).

An important component of validity studies is testing the invariance of the measurement instrument with respect to the variables which may be relevant for theoretical, ethical, or legal reasons. For these reasons, gender is one of the variables most commonly studied. In the case of the TEC, it should be ensured that a boy and a girl with the same level of emotion comprehension have the same probability of answering the test items correctly. If the items of the test do not comply with said invariance, we say that there is differential item functioning. The existence of differences between groups, which technically is called impact, should not be confused with DIF. DIF indicates a difference in item performance between boys and girls who have the same level of emotion comprehension, whatever the distribution of the ability between the groups. To the extent that the total score on the test is usually the sum of the scores of the items which comprise it, a large number of items with DIF against one group lead to scores which systematically undervalue this group. If we use this test to compare groups, the differences found might not correspond to real differences in the distribution of ability among groups.

There is an extensive corpus of psychometric research on the best statistical procedures for detecting DIF (for a review see Osterlind and Everson (2009); Penfield and Camilli (2007). When the response to items is dichotomous (right/wrong or pass/fail), the sample size is small (*N* < 250 per group), and the DIF is uniform (the item favours the same group on all levels of the construct measured), the method of reference is the Mantel–Haenszel (MH) procedure. A limitation of this procedure is its inability to detect some types of non-uniform DIF (the item favours a group on low ability levels and is detrimental at high levels, and the opposite with the other group). Thus, it is recommended that the analysis is complemented with logistic regression, which is sensitive to non-uniform DIF. Given that the majority of research on emotion comprehension in children has relied on small sample sizes, the techniques mentioned above are the methods of choice in this field.

Once the TEC has been analysed for DIF, we are then able to examine whether there are differences between boys and girls in the different measures of emotion understanding provided by the TEC. Some studies which have used other measures of emotion understanding have indeed found differences in favour of girls (Bajgar et al. (2005); (Bosacki and Moore 2004). However, most of the studies that use the TEC have not found statistically significant differences between boys and girls (Aldrich et al. 2011; Aznar and Tenenbaum 2013; Belacchi and Farina 2010; Farina and Belacchi 2014; Grazzani and Ornaghi 2012; Molina et al. 2014; Morra et al. 2011; Pons et al. 2004; Pons et al. 2002; Pons and Harris 2005; Pons et al. 2003; Pons et al. 2014; Tenenbaum et al. 2004). The majority of the cited studies used the total TEC score as the dependent variable and model-based methods for testing statistical significance. In contrast, this study will use the TEC components as the units of analysis because the differences in gender at the component level could be masked when using the total score (which is the result of the sum of all the components) as the dependent variable. Moreover, we will use a randomization-based method for testing statistical significance.

In sum, there are no studies evaluating whether tests used to evaluate emotion comprehension are invariant with respect to a child’s gender. To fill this gap in the literature, the present study examines whether there are gender differences in the different components of the most popular tests assessing emotion understanding in children. More specifically, we use the Mantel–Haenszel and logistic regression to examine whether there are gender differences in DIF.

## Method

### Participants

The participants of the present study were 353 typically developing children (181 boys and 172 girls), ranging from 3 to 8 years (*M*_boys_ = 5.17, SD = 1.65; *M*_girls_ = 5.16, SD = 1.56), from a number of playgroups, nurseries, and primary schools in the greater London, UK area and surrounding counties. They all lived within 1 h by train (up to 60 miles) of London. They were of broadly middle-class backgrounds (lower to upper-middle class). Table 1 describes the sample in terms of gender and age groups.

**Table 1:** Distribution of the sample in terms of gender and age (*N* = 353)

	Gender		
Age (in years)	boys	girls	Total
3	42	38	80
4	32	24	56
5	19	31	50
6	43	42	85
7	31	26	57
8	14	11	25
Total	181	172	353

Participants were recruited on a volunteer basis. All parents signed an informed consent form.

### Procedure

The TEC was administered in a quiet room in the schools and nurseries by a trained researcher. Its administration typically lasted 10 min.

### Measures

Participants’ responses to the TEC can be scored in at least three ways. First, they can be scored according to its nine components. A maximum of 1 point is provided for each component. Components I (recognition) and II (external cause) are comprised of five questions. Children receive a 1 on these two components if they answer four items out of five correctly. Components III (desire) and IX (moral) are comprised of two questions and children must answer both questions correctly to receive a 1 on these components. All the other components are represented by one question that is scored as pass or fail. Second, the TEC can be scored according to its subscales. The score obtained in each subscale ranged from 0 to 3, and is calculated by summing the scores obtained in each component belonging to the subscale. The external subscale includes the three first components: recognition, external cause, and desire. The mental subscale includes the next three components: belief, reminder, and regulation. The reflective subscale includes the last three components: hiding, mixed, and morality. Participants were given a pass–fail classification for each subscale. The subscales are scored as passed when all the components of the set are correctly answered. Otherwise, the subscale is scored as failed. The third way of scoring the TEC is using its total score. The overall level of emotion understanding in the TEC is calculated by summing the 9 components correctly answered. Thus, the total scale score range from 0 to 9. For a detailed description of the test and its scoring rules, see (Pons et al. 2004).

### Data Analyses

#### Testing DIF. Mantel–Haenszel procedure (MH)

As mentioned in the introduction, the DIF detection methods should make comparisons between the groups comparing individuals on the same level in the construct measured so as not to confuse impact with DIF. The MH procedure usually uses the total score as an estimate of the construct measured by the test. Therefore, the total TEC score is the stratification variable used to make the necessary group comparison (reference group = girls/focal group = boys). The logic behind the MH procedure is simple: If the variables group and response were independent, the odds of the probability of correctly responding to the item (*π*) instead of incorrectly (1-*π*) would be equal in the reference and focal groups. That is,

1 $$\frac{{\pi _{\rm R}}}{{1 - \pi _{\rm R}}} = \frac{{\pi _{\rm F}}}{{1 - \pi _{\rm F}}}$$

The above equality can be expressed as a ratio such that the ratio of the odds, referred to as the odds ratio, will be 1. Assuming homogeneity of the odds ratios of each stratum, the MH measure of association is the common odds ratio estimator ($\hat \alpha _{{\rm MH}}$). $\hat \alpha _{{\rm MH}}$ can be used as a measure of DIF effect size in a metric that varies between 0 and ∞. A value of 1 indicates independence between rows and columns (No DIF). $\hat \alpha _{{\rm MH}}$ > 1 indicate DIF in favour of the reference group (girls) and $\hat \alpha _{{\rm MH}}$ < 1 indicate DIF in favour of the focal group (boys).

Holland and Thayer (1988) proposed the MH chi-square statistic, $\chi _{{\rm MH}}^2$, (Mantel and Haenszel (1959) to test the null hypothesis of no DIF ($\alpha _{{\rm MH}}$ = 1). The $\chi _{{\rm MH}}^2$ statistic follows a chi-squared distribution with one degree of freedom. Simulations studies suggest that the $\chi _{{\rm MH}}^2$ statistic without the continuity correction tends to be less conservative than with the continuity correction (Paek (2010). For this reason we will compute $\chi _{{\rm MH}}^2$ omitting the continuity correction.

In order to assess and identify DIF items the Educational Testing Service (ETS) DIF classification criteria will be used (Zwick (2012)). The categorical rating of the severity of DIF is based on both the statistical significance of the results and the size of the effect. Because of the skewness of the distribution of $\hat \alpha _{{\rm MH}}$, it is more convenient to use the natural logarithm of $\hat \alpha _{{\rm MH}}$ $\left[ {\hat \lambda _{{\rm MH}} = ln(\hat \alpha _{{\rm MH}})} \right]$_._ According to this classification,

DIF is negligible if ${\mathrm{\lambda }}_{{\rm MH}}$is not significantly different from 0 (*p* ≥ .05) or $\left| {\hat \lambda _{{\rm MH}}} \right| < 0.426$.

DIF is moderate if *λ*_MH_ is significantly different from 0 (*p* < .05) and $\left| {\hat \lambda _{{\rm MH}}} \right| \ge 0.426$ and either: (a) $\left| {\hat \lambda _{MH}} \right| < 0.638$, or (b) *λ*_MH_ is not significantly greater than 0.426 (*p* ≥ .05).

DIF is large if $\left| {{\mathrm{\lambda }}_{{\rm MH}}} \right|$ is significantly greater than 0.426 (*p* < .05) and $\left| {\hat \lambda _{{\rm MH}}} \right| \ge 0.638$.

A modification of the GMHDIF program (Fidalgo 2011a, b) was used to compute all the MH statistics.

#### Testing DIF. Logistic regression (LR)

LR was first proposed for detecting DIF by (Swaminathan and Rogers 1990). It assesses to what extent item scores (1 correct response, 0 incorrect response) can be predicted from total scores alone (No DIF, model 1), from total scores and group membership (uniform DIF, model 2), or from total scores, group membership, and interaction between total scores and group membership (non-uniform DIF, model 3).

$$ln\left( {\frac{p}{{1 - p}}} \right) = \beta _0 + \beta _1X\quad \quad (model\,1)$$

$$ln\left( {\frac{p}{{1 - p}}} \right) = \beta _0 + \beta _1X + \beta _2G\quad \quad ({\it{model}}\,2)$$

$$ln\left( {\frac{p}{{1 - p}}} \right) = \beta _0 + \beta _1X + \beta _2G + \beta _3XG\quad \quad (model\,3)$$

In our case, *ln* is the natural logarithm, *p* is the probability of correct response to the studied component, *X* is total TEC scores, G is a dummy variable representing group membership (1 = reference group/girls, 0 = focal group/boys), *XG* is the interaction term between total TEC scores and group membership, and *β*s are the parameters in the model. The strategy for evaluating the DIF is based on the search for the most parsimonious model that best fits the data. To use LR for DIF analysis, Models 1, 2 and 3 were fit to the data using the SPSS (version 18).

LR also gives an estimation of the magnitude of uniform DIF, the $\hat \beta _{\rm 2}$ coefficient calculated in the model 2. The criteria for assessing the severity of DIF are the same as for the MH procedure, because $\hat \lambda _{{\rm MH}}$ and $\hat \beta _{\rm 2}$ are equivalent. That is, the ETS DIF classification system described above was applied (for more detailed information see, Monahan et al. (2007)).

This study employs an additional measure of the magnitude of DIF based on Nagelkerke’s R^2^. This measure enables both the magnitude of uniform and non-uniform DIF to be estimated. Thus non-uniform DIF is equal to the difference in Nagelkerke’s *R* ^2^ between the non-uniform and uniform DIF models: $\Delta R_{\rm N}^2 = R^2\left( {model\,3} \right) - R^2\left( {model\,2} \right)$. And uniform DIF is equal to: $\Delta R_{\rm U}^2 = R^2\left( {model\,2} \right) - R^2\left( {model\,1} \right)$. The guidelines proposed by (Jodoin and Gierl 2001) to quantify the magnitude of DIF are as follows:

Negligible DIF: Δ*R* ^2^ < 0.035

Moderate DIF: 0.035 ≤ Δ*R* ^2^ ≤ 0.070

Large DIF: Δ*R* ^2^ > 0.070

Following the criteria of Jodoin and Gierl (2001), an item is considered to have DIF if the probability of either 1 − d*f* *χ* ^2^ test was less than .05, and the corresponding Δ*R* ^2^ ≥ .035.

The reader can found a detailed description of the LR for DIF analysis in Fidalgo et al. (2014).

#### Testing gender differences

The $\chi _{{\rm MH}}^2$statistic (Mantel and Haenszel (1959) and the Mantel test (Mantel 1963) were employed to examine whether there are statistically significant differences between boys and girls in the different measures of emotion comprehension provided by the TEC, while controlling for age. To do so, the responses on the TEC (response variable) of girls and boys (factor) were compared within the same age group (stratification variable or covariate). The null hypothesis (*H*_0_) they test establishes that, in each one of the strata of the covariable (age), the response variable (TEC scores) is distributed randomly, with respect to the gender of the children. That is, the answers on the TEC are independent of the child’s gender.

The analysis was conducted by applying the $\chi _{{\rm MH}}^2$statistic to dichotomous scores, such as the components or subscales scored as a pass–fail classification. The $\chi _{{\rm MH}}^2$ statistic follows a chi-squared distribution with one degree of freedom. When the response variable has more than two categories and is measured on an ordinal scale, the pertinent statistic is the Mantel Test. Under *H*_0_, the Mantel test has approximately a chi-squared distribution with d*f* = (*R* − 1), being *R* the number of groups. The choice of statistics included in the MH methodology, instead of an analysis of covariance (ANCOVA), which would be the most common parametric alternative, is determined by the non-randomized nature of the sample available. The model based methods, like ANCOVA, requires that participants constitute a random sample of subjects from a well-defined population (Manly 2006; Zheng and Zelen 2008). Unfortunately, that is a very unrealistic assumption in this field of research. On the contrary, MH statistics permit the use of samples of convenience on not assuming a known sampling link to a larger reference population (Koch et al. 1980). This is possible, thanks to the fact that the *H*_0_ of interest—that the distribution of the responses is random with respect to the levels of the factor—induces a probabilistic structure (the multiple hypergeometric distribution) that allows for judgment of its compatibility with the observed data without the need for external assumptions. More detailed information about this methodology and its use in the behavioral sciences can be found in Fidalgo (2005).

In addition to determining statistical significance, measures of effect size were used to evaluate the extent of the association between gender and the responses on the TEC. In the case of dichotomous responses,$\hat \alpha _{{\rm MH}}$, was used as described in the section on *Testing DIF*. When the response variable has more than two categories, the pertinent statistic is the Liu-Agresti estimator of the cumulative common odds ratio statistic ($\hat \psi _{{\rm LA}}$) (Penfield and Algina 2003). It should be note that $\hat \psi _{{\rm LA}}$ is a generalization of $\hat \alpha _{MH}$ for this case (Liu and Agresti 1996).

## Results

The first psychometric property of the TEC evaluated was its internal consistency, which had a Cronbach’s alpha of .66. Next, the DIF analyses were conducted. Table 2 shows $\chi _{{\rm MH}}^2$ statistics and related effect size measure ($\hat \alpha _{{\rm MH}}$), along with the results derived from the ETS DIF classification. As it may be observed, none of the TEC components functions differentially by gender. Results were identical when the LR was applied for detecting uniform and non-uniform DIF (see Table 3). None of the components showed DIF, by either the ETS system classification or the criteria proposed by Jodoin and Gierl (2001).

**Table 2:** Summary of the Mantel–Haenszel gender DIF analyses for the TEC components

TEC Component	$\chi _{{\rm MH}}^2$	*p-*value	$\hat \alpha _{{\rm MH}}$	ETS DIF classification
Recognition	0.275	.600	1.330	Negligible DIF
External cause	0.047	.828	1.073	Negligible DIF
Desire	2.328	.127	0.642	Negligible DIF
Belief	1.514	.218	1.333	Negligible DIF
Memory	0.702	.402	0.805	Negligible DIF
Regulation	0.640	.424	1.242	Negligible DIF
Hiding	0.181	.670	0.894	Negligible DIF
Mixed	0.223	.637	0.874	Negligible DIF
Morality	0.432	.511	1.231	Negligible DIF

$\chi _{{\rm MH}}^2$: MH chi-square statistic used to test the null hypothesis of No DIF (*H*_0_: *α*_MH_ = 1). This statistics follows a chi-squared distribution with one degree of freedom

$\hat \alpha _{{\rm MH}}$: MH common odds ratio estimator. $\hat \alpha _{{\rm MH}}$ > 1 indicate DIF in favour of the reference group (girls) and $\hat \alpha _{{\rm MH}}$ < 1 indicate DIF in favour of the focal group (boys)

ETS DIF classification: Classification of DIF based on the criteria proposed by the Educational Testing Service (ETS): negligible DIF/ moderate DIF/large DIF

There was no necessary to purify total test scores given that none component was identified displaying DIF in the first analysis

**Table 3:** Summary of the Logistic Regression DIF analyses for the TEC components

						DIF classification criteria	
Component	*H*_0_ Hypotheses	$\hat \beta$	Wald chi-square	*p-*value	Δ Nagelkerke *R* ^2^	Jodoin and Gierl (2001)	ETS
Recognition							
	No non-uniform DIF	−0.434	0.619	.431	0.004	Negligible DIF	–
	No uniform DIF	0.283	0.250	.617	0.002	Negligible DIF	Negligible DIF
External cause							
	No non-uniform DIF	−0.055	0.027	.869	0.000	Negligible DIF	–
	No uniform DIF	−0.100	0.081	.776	0.000	Negligible DIF	Negligible DIF
Desire							
	No non-uniform DIF	0.340	2.556	.110	0.007	Negligible DIF	–
	No uniform DIF	−0.382	1.796	.180	0.005	Negligible DIF	Negligible DIF
Belief							
	No non-uniform DIF	0.235	3.169	.075	0.010	Negligible DIF	–
	No uniform DIF	0.393	2.841	.092	0.009	Negligible DIF	Negligible DIF
Memory							
	No non-uniform DIF	0.248	1.909	.167	0.006	Negligible DIF	–
	No uniform DIF	−0.216	0.660	.416	0.002	Negligible DIF	Negligible DIF
Regulation							
	No non-uniform DIF	−0.274	1.905	.168	0.005	Negligible DIF -	
	No uniform DIF	0.393	2.063	.151	0.005	Negligible DIF	Negligible DIF
Hiding							
	No non-uniform DIF	−0.366	3.314	.069	0.008	Negligible DIF	–
	No uniform DIF	−0.053	0.037	.848	0.000	Negligible DIF	Negligible DIF
Mixed							
	No non-uniform DIF	−0.243	1.085	.298	0.003	Negligible DIF	–
	No uniform DIF	0.094	0.103	.748	0.000	Negligible DIF	Negligible DIF
Morality							
	No non-uniform DIF	−0.264	1.506	.220	0.006	Negligible DIF	–
	No uniform DIF	0.486	2.400	.121	0.009	Negligible DIF	Negligible DIF

*H*_0_ Hypotheses: No non-uniform DIF (*H*_*o*_: *β*_*3*_ = 0 (Model 3)). No uniform DIF (*H*_*o*_: *β*_*2*_ = 0 (Model 2))

$\hat \beta :\hat \beta$ coefficient calculated in the LR model 3 ($\hat \beta _{\rm 3}$) and LR model 2 ($\hat \beta _{\rm 2}$). $\hat \beta _{\rm 2}$> 0 indicate DIF in favour of the reference group (girls), and $\hat \beta _2$< 0 indicate DIF in favour of the focal group (boys)

Wald chi-square: Wald statistic used to test the corresponding null hypotheses. That statistic follows a chi-squared distribution with one degree of freedom

Δ Nagelkerke *R* ^*2*^: Measure of the magnitude of DIF based on Nagelkerke’s *R* ^*2*^

DIF classification criteria: Classification of DIF based on the criteria proposed by Jodoin and Gierl (2001) and the Educational Testing Service (ETS): negligible DIF/ moderate DIF/ large DIF

This results have been obtained using the purified total test score (second stage). The total test score for each examinee was refined by removing the component belief that was found to show DIF in the first stage (−2 log likelihood [model 3-model 1] = 6.125171, *df* = 2, *p* = .047)

The results of the analysis of distribution of TEC scores are presented below (see Table 4). On the total test score level, we found statistically significant differences in favour of girls (Mantel test = 7.207, *p* = .007, $\hat \psi _{{\rm LA}} =$ 1.691). In the analysis of subscales, we only found differences in the mentalistic subscale. On the component level, we only found statistically significant differences in the Belief component. When the effect size was evaluated, it was found that the odds of answering correctly the belief component is estimated to be 1.75 times greater for girls than boys, adjusting for age. If we reanalyse the mentalistic subscale, eliminating the belief component from the calculation, there are no longer any statistically significant differences between boys and girls, whether scoring on the 0 to 2 scale (Mantel test = 1.343, *p* = .247, $\hat \psi _{{\rm LA}}$ = 1.286) or dichotomously ($\chi _{{\rm MH}}^2$= 1.06, *p* = .301, $\hat \alpha _{{\rm MH}} = 1.318$). Equally these differences decrease, although they remain statistically significant (*α* = .05), when the belief component is eliminated from the total TEC score (Mantel test = 3.897, *p* = .048, $\hat \psi _{{\rm LA}}$ = 1.464). It may therefore be concluded that the belief component is largely responsible for the differences between boys and girls in the TEC scores.

**Table 4:** Results of the gender difference analysis with Mantel–Haenszel methods

TEC Scores	MH statistic	*p-*value	Effect size statistic
Components	$\chi _{{\rm MH}}^2$	*p-*value	$\hat \alpha _{{\rm MH}}$
Recognition	2.640	.104	2.265
External cause	0.799	.371	1.325
Desire	0.151	.698	0.904
Belief	6.406	.011	1.750
Memory	0.000	.991	0.997
Regulation	2.525	.112	1.459
Hiding	0.493	.483	1.188
Mixed	0.674	.412	1.221
Morality	3.670	.055	1.749
Subscales (scored pass or fail)	$\chi _{{\rm MH}}^2$	*p-*value	$\hat \alpha _{{\rm MH}}$
External	0.304	.581	1.158
Mental	6.487	.011	2.238
Reflective	3.142	.076	2.067
Subscales (scored 0–3)	Mantel Test	*P-value*	$\hat \psi _{{\rm LA}}$
External	0.682	.409	1.220
Mental	6.417	.011	1.686
Reflective	3.158	.076	1.438
Total TEC scores	7.207	.007	1.691

MH statistic: MH statistics used to test the null hypothesis of independence between TEC scores and gender, controlling by age. $\chi _{{\rm MH}}^2$ and the Mantel test. In our case, both statistics follow a chi-squared distribution with one degree of freedom

Effect size statistic: MH statistics to estimate the effect magnitude $\hat \alpha _{{\rm MH}}$: MH common odds ratio estimator. $\hat \psi _{{\rm LA}}$: Li-Agresti estimator of the cumulative common odds ratio. In both estimators values >1 indicate advantage of the reference group (girls) and values <1 indicate advantage of the focal group (boys)

## Discussion

Developed by the International Test Commission (ITC), the International Guidelines for Test Use are a set of guidelines that provide an international view on what constitutes “good practice” in test use. In Section 2.3 on issues of fairness in testing, the ITC recommends the need of DIF studies when tests are to be used with individuals from different groups (International Test Commission 2001). In fact, the study of differential item functioning is one of the routine stages in the construction and evaluation of tests in aptitude and educational testing. Unfortunately, in other areas of psychology, DIF analyses between groups that are subject to frequent comparison are not common. This is the case, for example, of the tests designed to evaluate emotion comprehension in children, and more specifically, of the TEC. Therefore, the first goal of this study was to determine whether the TEC components display gender DIF. The results indicate that none of the nine components of the TEC function differentially in boys and girls. That is, children with the same level of emotion comprehension have the same probability of passing the component, regardless of their gender.

Next, we examined whether there are differences between boys and girls in the different measures of emotion comprehension provided by the TEC. To date, the study of gender differences has always been a secondary goal of studies employing the TEC. Furthermore, these studies have typically used the total TEC score as the dependent variable. When the subscales were analysed, we found statistically significant differences only in the Mentalistic subscale. An individual analysis of the various components showed that the cause of the differences between boys and girls on this subscale was due exclusively to the Belief component (see Table 4). Similarly, the belief component is largely responsible for the differences between boys and girls in the total TEC scores.

**Fig. 1:**
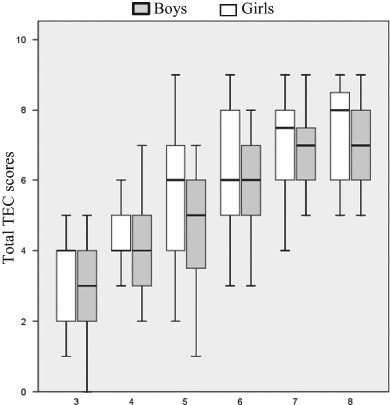
Box-Plot with the total TEC scores distribution by age and gender. Age (years). The lower boundary of the box is the 25th percentile, and the upper is the 75th; the horizontal bold line inside the box represents the median value; vertical lines out of the box indicate the range of scores. Total test score grew with age, but on average girls outperformed boys

There are several hypotheses that could explain the differences found. The first, and most general, is that girls have slightly earlier neurocognitive maturation that may serve ToM development which is at the base of much emotion comprehension (Thompson and Thornton 2014). In ToM studies reporting gender differences, the results have typically favoured girls (Calero et al. 2013; Devine and Hughes 2013). And more specifically, some research has shown better emotion comprehension by girls (Bajgar et al. 2005; Bosacki and Moore 2004), which is in accordance with the results found here (see Table 4 and Fig. 1).

This hypothesis of maturational differentiation would explain the small differences in favour of females in the total TEC score found across all ages. However, it would not explain why this difference is only statistically significant and of a relevant magnitude for the belief component. The second explanation is much more specific and has to do with the differences between boys and girls in cognitive knowledge of false belief. In the TEC (Pons et al. 2004), children are first asked about a rabbit who cannot see a fox behind a bush. After being asked if the rabbit cannot see the fox (and being corrected if they are incorrect), children are asked how the rabbit feels. As accurately described by Morra et al. (2011), “the component ‘Belief’ of the TEC is similar to a classical false-belief task, because it involves (a) an element of factual information and (b) a representation of the protagonist’s state-of-knowledge, but in addition, the rabbit/fox problem also involves a third element (c) that represents the affective value of state (a) for the protagonist”. It seems that the attribution of emotions based on false beliefs is a task which is acquired later than cognitive knowledge of false belief (Bradmetz and Schneider 1999; de Rosnay et al. 2004), and that can be partially explained in terms of a differential working memory load (Morra et al. 2011). As Harris (2008) argues, to pass false belief on this task, one must set aside knowledge of imminent danger. Given boys’ greater propensity for crying at a young age (Weinberg 1992), this finding suggests that boys continue to find it difficult to ignore knowledge of negative emotions. Nevertheless, the second hypothesis assumes the first hypothesis of brain maturational differences (Charman et al. (2002)).

### Limitations

This study introduces DIF as a necessary part of the study of TEC validity, and by extension, other tests and questionnaires designed to measure emotion comprehension. The data analysed are compatible with the hypothesis that the scores on the various TEC components are independent of the gender of the children evaluated. That is, that the TEC does not show Gender DIF. Methodologically, one of the limitations of our study is the use of age in years as the stratification variable. Clustering the children by age in years assumes that children who might be in different periods of maturation are grouped together. The use of months as a measure of age instead of years would no doubt increase the precision of the analyses.

These findings add to the accumulation of contradictory evidence in research on gender differences. If in the scope of expression of emotions there seem to be small but significant differences in gender (Chaplin and Aldao 2013) Chaplin 2015), in the field of emotion comprehension the evidence is not so clear. Our data are compatible with the hypothesis of independence between genders and level of comprehension in 8 of the 9 components of the TEC. Given that the Belief component is basically a false belief task, the differences found seem to support findings in the literature indicating that girls perform better on this task (Charman et al. 2002; Devine and Hughes 2013) rather than studies that do not find differences in gender (Hughes et al. 2011; Kolodziejczyk and Bosacki 2015). It should be stressed that the basis of our inferences is the randomization mechanism implicit in the MH tests and not random sampling from a target population. This study evaluated gender differences in emotion comprehension controlling for age. Other variables that might influence results, such as verbal ability or family characteristics (number of siblings, mother’s education) were not controlled for, and could act as confounding variables. In sum, our findings suggest that on the majority of components of emotion understanding, boys’ and girls’ understanding is more similar than different.
